# Universal Southern blot protocol with cold or radioactive probes for the validation of alleles obtained by homologous recombination

**DOI:** 10.1016/j.ymeth.2020.06.011

**Published:** 2020-06-26

**Authors:** Gemma F. Codner, Valerie Erbs, Jorik Loeffler, Lauren Chessum, Adam Caulder, Nicolas Jullien, Sara Wells, Marie-Christine Birling, Lydia Teboul

**Affiliations:** a The Mary Lyon Centre, MRC Harwell Institute, Harwell Campus, Didcot, Oxon OX11 0RD, UK; b PHENOMIN-Institut Clinique de la Souris, CNRS, INSERM, Université de Strasbourg, Illkirch-Graffenstaden, Strasbourg 67404, France; c Aix-Marseille University, CNRS, INP, Institut de Neurophysiopathologie, Marseille, France

**Keywords:** Southern blot, Homologous recombination, Targeting, Validation, Mouse

## Abstract

The widespread availability of recombineered vectors and gene targeted embryonic stem cells from large-scale repositories facilitates the generation of mouse models for functional genetic studies. Southern blotting validates the structure of these targeted alleles produced by homologous recombination, as well as indicating any additional integrations of the vector into the genome. Traditionally this technique employs radioactively-labelled probes; however, there are many laboratories that are restricted in their use of radioactivity. Here, we present a widely applicable protocol for Southern blot analysis using cold probes and alternative procedures employing radioactive probes. Furthermore, the probes are designed to recognise standardised regions of gene-targeting cassettes and so represent universally applicable reagents for assessing allelic integrity.

## 1. Introduction

Gene targeting in embryonic stem (ES^1^) cells typically requires antibiotic selection to the isolate-resistant colonies, which are then screened with a rapid and inexpensive protocol, often PCR-based, to confirm the targeting event. Subsequently, positive colonies, in which on-target modification is identified, must be further characterised to complete validation of the new alleles. Southern blot-based methods can be employed to report on the structure of the targeting events and the number of integrations of the targeting construct. However, it frequently requires a new set of probes specific to each targeting project, for which experimental conditions must be established or adapted.

Conditional and other complex genome-engineered alleles are essential tools for functional genetics. As part of the International Mouse Phenotyping Consortium (IMPC), we have been involved in a large-scale effort to generate mouse mutants from a vast collection of ES cell clones containing knockout-first alleles [1]. As part of the ES cell to mouse conversion pipeline, we have developed a generic protocol to validate the integrity of targeted alleles in ES cells and mouse tissues using Southern blotting and non-radioactive probes [2].

The principle of Southern blotting [3] is based upon the ability to separate DNA fragments obtained by restriction digestion according to their size by gel electrophoresis [4] and, classically, on the hybridization of radioactively-labelled probes to nucleic acids that are denatured and immobilised on nylon or nitrocellulose membranes [5]. Southern blotting was applied to monitor the structure of targeted mutations in ES cells [6]. Segments corresponding to the genomic sequences that are adjacent to each homology arm but are not contained in the targeting construct are defined as external probes and have been employed to validate homologous recombination events [7]. Additionally, external probes also allow visualisation of the wild type allele. By contrast, here we employ sequences that are contained within the targeting construct [7] and are defined as internal probes. Using either internal or external probe approaches, the presence of a single or a second positive band of the expected electrophoretic mobility informs on a homologous recombination event, respectively. More recently, we have adopted the use of digoxigenin-labelled probes and enzymatic reactions as an alternative to radioactive chemistry [8].

For this process, genomic DNA (gDNA) is digested by restriction enzymes, generating fragments of a given size that are separated by gel electrophoresis. The DNA fragments are denatured, transferred and immobilised onto nylon membranes. Probes identify bands containing the target sequences. The number and size of bands can be measured against size-standard ladders and compared to expected values.

Here, we present a protocol that employs internal probes that recognise either the *lacZ* reporter or the neo selectable marker sequence which are common to all of the targeted alleles that we have validated as part of our IMPC ES cell to mouse conversion pipeline. This protocol can be adapted for validation of targeted events in any other homologous recombination experiments.

## 2. Material and methods

### 2.1. Extraction of gDNA

Reagents:

1 M Tris-HCl pH 7.5

0.5 M EDTA

20% SDS

5 M NaCl

10 mg/ml Proteinase K

Phenol, pH7.9:chloroform:isoamyl alcohol (IAA) 25:24:1. Note that this is a hazardous chemical. Appropriate precautions (as per applicable regulations) must be taken at all steps involving this compound.

Phase Lock Gel Tubes (Five Prime, 2302820)

Isopropanol

Pure ethanol

MilliQ water

gDNA extraction stock solutions:

Prepare a stock of Lysis buffer ensuring the water is added first, followed by the remaining reagents (50 mM Tris-HCl, pH 7.5, 5 mM EDTA, 2% SDS, 200 mM NaCl). Mix well by inverting and store at room temperature. Prepare 10 mg/ml Proteinase K and aliquot; store at −20 °C.

#### 2.1.1. Extraction of gDNA from cultured ES cell pellets

ES cell samples are collected from an overconfluent 9 cm diameter plate; trypsinise ES cells and pellet into an Eppendorf tube. Alternatively, gDNA can be extracted directly from cell plates.

Day 1: Prepare a PK Lysis mix by adding 75 μl 10 mg/ml Proteinase K to 750 μl Lysis buffer, scaling up according to the number of samples. Add 825 μl PK Lysis mix to each well or tube containing an ES cell sample. Using a wide-bore tip, gently pipette up and down to mix cell pellet and lysis buffer. If extracting directly from cell plates, wet a piece of kitchen paper and place in the base of a plastic box. Seal around the lid of each ES cell plate with parafilm to prevent the samples drying out and place into the plastic box. Incubate samples with the PK Lysis mix overnight at 55 °C.

Day 2 : If extracting from a plate, using a wide-bore tip, vigorously scrape each well to remove any ES cells adhering to the plate and transfer the sample to a fresh Eppendorf. Before adding phenol, mix the lysis solution up and down with a wide-bore tip to remove residual viscosity. In a fume hood, add one volume (~600 μl) of phenol:chloroform:IAA (avoid the top aqueous layer and use only the lower phase) and shake vigorously. Do not vortex. Tape the sample tubes onto a rocker and mix by rocking at low speed for 15 min. In a fume hood, pour into a Phase Lock Gel Tube and spin in a microcentrifuge at full speed for 5 min. This will separate the sample into an upper aqueous phase containing the gDNA and a lower organic phase containing the phenol:chloroform:IAA solution. Collect the upper aqueous phase by pouring into a new Eppendorf and discard the lower phase. Add 700 μl isopropanol and mix well by inversion. This will precipitate the DNA into a spider-web-like structure. Continue inverting until the precipitate is compact/clearly visible. Spin at full speed for at least 15 min to pellet the precipitated DNA. Carefully remove the supernatant. Wash the pellet by adding 200 μl 70% ethanol. Spin for 1 min then remove the ethanol. Air dry the pellet for 15–20 min by leaving the tube open on the bench. Gently resuspend the pellet in 400 μl MilliQ water. Correlate the amount of water to the size of the pellet with roughly 100 μl per mm of pellet diameter. Handle gently as the DNA will be viscous. Always use cut or wide-bore tips when pipetting gDNA to avoid shearing and damaging the DNA. Store at 4 °C. Resuspend at least overnight, preferably over a couple of days. Before quantification, it is very important to gently pipette up and down with a wide-bore tip. This ensures homogeneity of the DNA solution and avoids inaccurate spectrophotometer readings. In addition, if the solution is very glutinous more water can be added, again followed by gentle mixing with a wide-bore tip. Quantify using Nanodrop or other spectrophotometry-based method. Adjust the volume of water so that the DNA concentration is not > 1 μg/μl.

#### 2.1.2. Extraction of gDNA from soft tissues

gDNA is extracted as in 2.1.1 with the following modifications.

Additional equipment:

Purple gentleMACS C-Tube (Miltenyi Biotec) or other mechanical or hand-held homogenisation device

Day 1: Cut spleen in half using dissecting scissors (or dissect a piece of liver of a similar size). Place one half into an Eppendorf and freeze for future use. Place the tissue sample into a purple gentleMACS C-Tube and add 1.5 ml PBS. Homogenise the tissue using the gentleMACS program *m-spleen-04.01*. Repeat homogenisation using the same program for a second time, checking for remaining tissue that may be stuck in the blades of the tube lid. With a wide-bore tip, pipette the mixture into an Eppendorf and spin at full speed for 5 min to pellet the sample. Remove the PBS and add 750 μl of Lysis buffer. Using a wide-bore tip, thoroughly mix the tissue in the Lysis buffer. Add 75 μl of 10 mg/ml Proteinase K. Using a wide-bore tip, gently pipette the whole volume up and down to mix. Incubate at 55 °C for at least 3–4 h. Before the end of the day, add a further 200 μl of Lysis buffer and 40 μl of 10 mg/ml Proteinase K to the spleen (or liver) sample. Using a wide-bore tip, mix up and down. Incubate overnight at 55 °C.

Day 2: The next morning, add a further 200 μl of Lysis buffer and 40 μl of 10 mg/ml Proteinase K to each sample. Use a wide-bore tip to mix the whole volume up and down and incubate at 55 °C for another 3–4 h. Lysis is complete when the solution is homogenous. Even though spiking twice within 12 h is usually sufficient, spiking should be repeated/continued until homogeneity is achieved. After incubation of the tissue, separate the sample by pipetting two equal amounts (approximately 600 μl each) into two fresh Eppendorfs. In the fume hood, add one volume (approximately 600 μl) of phenol:chloroform:IAA to each sample and shake vigorously. Do not vortex. Tape Eppendorfs onto a see-saw rocker and mix by rocking at a low speed for 15 min. In the fume hood, pour the entire volume of each Eppendorf into a Phase Lock Gel Tube. Spin at full speed for 5 min. This will separate the samples into an upper aqueous phase containing the DNA and a lower organic phase containing chloroform. Combine the upper aqueous phases of the two Phase Lock Gel tubes per sample by carefully pipetting (using a wide-bore tip) about 400 μl together into one new Eppendorf. The lower phases can be discarded.

Add 700 μl isopropanol until the Eppendorf is nearly full and mix well by inversion. This will precipitate the DNA into a spider-web-type structure. Continue inverting the Eppendorf until the precipitate is compact. Spin at full speed for at least 15 min to pellet the precipitated DNA. Carefully remove the supernatant. Add 200 μl of 70% ethanol and spin for 1 min. Remove the ethanol. Air dry the pellet for 15–20 min by leaving the Eppendorf open on the bench. Gently resuspend in an appropriate amount of MilliQ water (correlate the amount of water to the size of the pellet). Handle gently, as the DNA will be stringy and glutinous. Store at 4 °C. Gently mix the solution and quantify using Nanodrop or other spectrophotometry-based method the following day. NOTE: Such concentrated gDNA solutions are challenging to homogenise. Optical density measurements only afford an approximative evaluation of DNA concentration.

#### 2.1.3. Extraction of gDNA from tail tissue

gDNA is extracted as in 2.1.1 with the following modifications.

Day 1: Cut the tail into small pieces by separating the vertebrae. Place 2–3 vertebrae pieces into an Eppendorf and store the remaining tail tissue in the freezer. NOTE: It is not necessary to homogenise tails. Add 750 μl of Lysis buffer and 75 μl of 10 mg/ml Proteinase K directly onto the sample and incubate overnight at 55 °C.

Day 2: The vertebrae bones and hairs of the tail will not digest. It is normal to see tissue debris. Spin the tail sample for 2 min at full speed to collect the debris at the bottom of the tube. Remove approximately 600 μl of the tail sample and place into one fresh Eppendorf. Add 600 μl of phenol:chloroform:IAA and shake vigorously (but do not vortex). Attach Eppendorfs onto a see-saw rocker and mix by rocking at a low speed for 15 min. In the fume hood, pour the entire volume of each Eppendorf into a Phase Lock Gel Tube. Spin at full speed for 5 min. This will separate the samples into an upper aqueous phase containing the DNA and a lower organic phase containing chloroform.

Transfer the upper aqueous phase into one new Eppendorf. The lower phases can be discarded. Add 700 μl isopropanol until the Eppendorf is nearly full and mix well by inversion. This will precipitate the DNA into a spider-web-type structure. Continue inverting the Eppendorf until the precipitate is compact. Spin at full speed for at least 15 min to pellet the precipitated DNA. NOTE: A DNA pellet might not be clearly visible.

Carefully remove the supernatant. Add 200 μl of 70% ethanol and spin for 1 min. Remove the ethanol. Air dry the pellet for 15–20 min by leaving the Eppendorf open on the bench. Gently resuspend in an appropriate amount of MilliQ water (correlate the amount of water to the size of the pellet). Handle gently, as the DNA will be stringy and glutinous. Store at 4 °C. Gently mix the solution and quantify using Nanodrop or other spectrophotometry-based method the following day.

NOTE: The amount of tissue required for extraction of sufficient material for the Southern blot process is larger than that obtained from tail biopsies that can be performed on live animals. However, tail samples are often available as frozen archive material and may represent a useful source of tissues for gDNA preparation.

### 2.2. Design of Southern assay

**Fig. 1.**
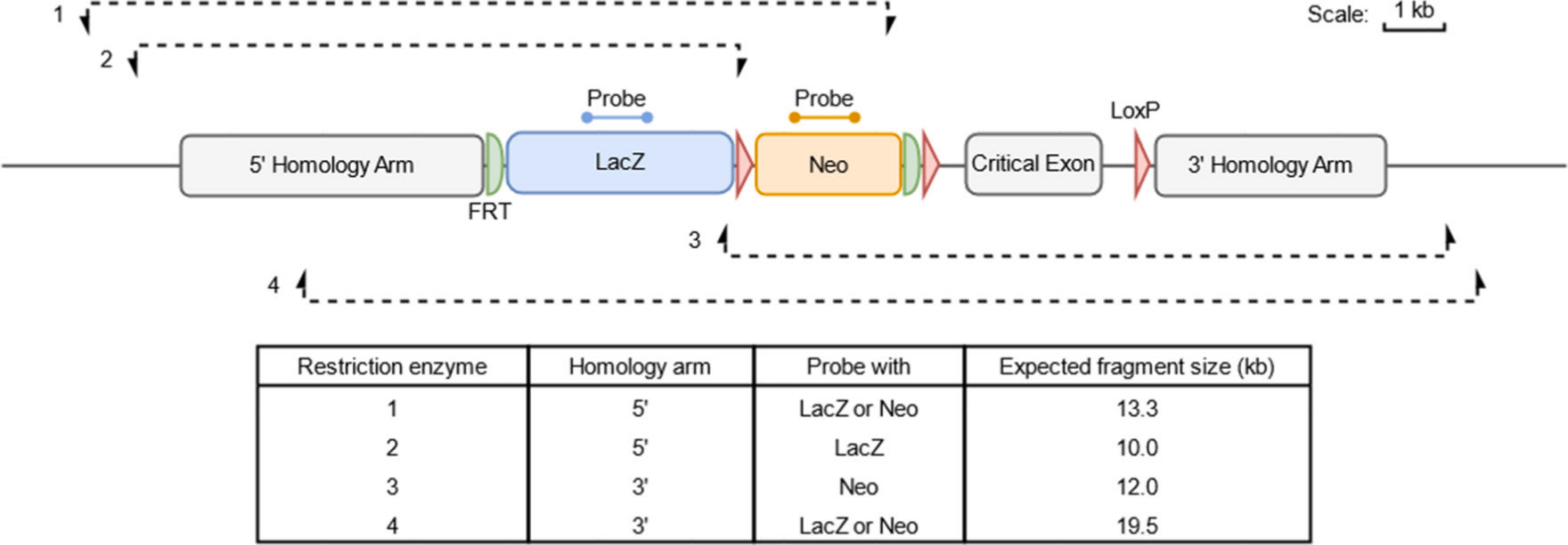
Design of Southern assay. The figure details a generic tm1a construct from the KOMP repository annotated with the positions of both the *lacZ* and neo probes used for Southern blot analysis. Four examples of restriction digests are annotated, which can be used to interrogate whether the cassette has integrated correctly on target into the genome; restriction digests using enzymes 1 and 2 are used to confirm correct insertion of the 5′ homology arm (HA) on target and restriction digests using enzymes 3 and 4 are used to verify the integrity of the 3′ HA. The expected sizes of the fragments of a correctly targeted allele and the probe(s) that can be used with each restriction digest are described in the table.

Restriction enzymes are selected per targeting experiment and should cut the genome to produce a fragment containing the sequence recognised by the probe, an entire homology arm and the genomic sequence flanking the targeting construct (Fig. 1). Digests are generally performed with a single enzyme, but it is sometimes necessary to combine two restriction enzymes in order to obtain a fragment of an acceptable size. Restriction enzymes should be chosen so that they generate a fragment of a size that can be resolved by migration in an agarose gel (fewer than 20 kb whenever possible; up to 24 kb with optimal electrophoresis conditions). At least two different digests for each homology arm are employed. Depending on the size of the generated fragment, a digest with only one enzyme that encompasses both homology arms is also possible. Enzymes inhibited by methylation in eukaryotic genomes are avoided whenever possible.

### 2.3. Synthesis of digoxigenin-labelled probes

Reagents:

DEPC-treated nuclease-free H_2_O (Ambion)

dATP, dCTP, dGTP, dTTP (all 100 mM: Thermo Fisher Scientific) DIG-dUTP (1 mM: Sigma)

OneTaq® DNA polymerase (New England Biolabs) or other standard Taq polymerase

*LacZ* primers 5′−3′: LacZF: TTGAAAATGGTCTGCTGCTG, LacZR: CGGATAAACGGAACTGGAAA

Neo primers 5′−3′: NeoF: GCTATTCGGCTATGACTGGG, NeoR: GAA GGCGATAGAAGGCGATG

Plasmid containing relevant cassettes (Sequences in Supplemental text 1)

QIAquick Gel Extraction Kit (Qiagen)

PCR is employed to incorporate DIG-labelled dUTP nucleotides into a PCR product, for use as a probe in cold Southern blot analysis. Any targeting vector from the EUCOMM or KOMP collection can be employed as a template for PCR. Alternatively, the sequences for the probes are specified in Supplemental text 1 and can be synthesized commercially and cloned for future use as a template.

Prepare the labelled dNTP mix by adding the following: 0.9 μl of each of dATP, dCTP and dGTP (all at 100 mM), 0.72 μl of dTTP (at 100 mM), 18 μl DIG-dUTP (at 1 mM) and 23.58 μl H_2_O, to a final volume of 45 μl. An equivalent unlabelled dNTP mix must also be prepared for use in a control reaction as follows: 0.9 μl of each of dATP, dCTP, dGTP and dTTP (all at 100 mM) and 41.40 μl H_2_O, to a final volume of 45 μl. Prepare a master mix of eight reactions for neo and *lacZ* probes with labelled dNTPs as follows and aliquot 50 μl per well: 80 μl 5x Buffer, 0.8 μl forward primer (100 μM), 0.8 μl reverse primer (100 μM), 40 μl dNTPs (2 mM, labelled), 268.4 μl H_2_O, 8 μl of plasmid containing the relevant cassette (500 ng/μl), 2 μl Taq polymerase. For each probe, prepare a control reaction with non-labelled dNTPs as follows: (per 50 μl reaction) 10 μl 5x Buffer, 0.2 μl forward primer (100 μM), 0.2 μl reverse primer (100 μM), 5 μl dNTPs (2 mM, unlabelled), 33.4 μl H_2_O, 1 μl of 500 ng/μl plasmid containing the relevant cassette, 0.25 μl Taq polymerase. NOTE: Do not use gDNA of a transgenic animal as a PCR template, as this would result in a large amount of background noise.

Run the PCR under the following conditions: 94 °C for 5 min, 42 cycles of 94 °C for 30 s, 58 °C for 30 s, 72 °C for 1 min, followed by 72 °C for 10 min, and then hold at 4 °C.

**Fig. 2.**
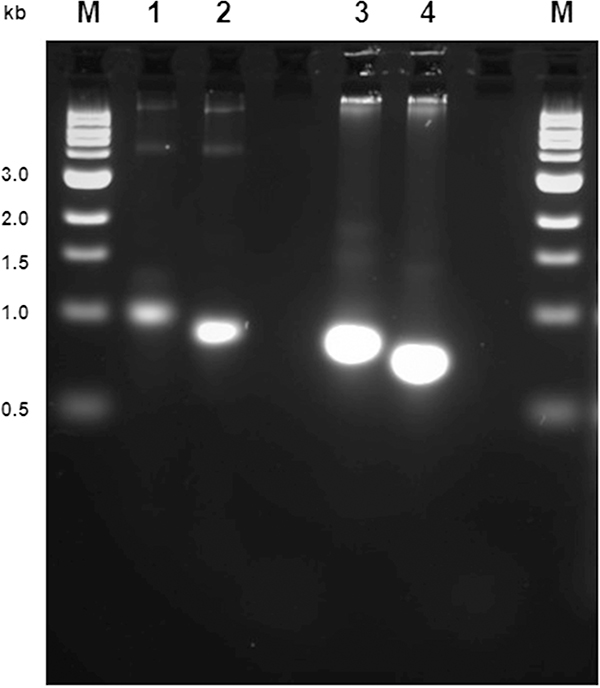
Analysis of DIG-labelled probe by agarose electrophoresis. M: molecular weight marker. *LacZ* (lanes 1 and 2) and neo (lanes 3 and 4) probes synthesised with (lanes 1 and 3) and without (lanes 2 and 4) DIG-labelled nucleotide. A difference in electrophoretic mobility must be observed.

To ascertain whether or not the template has been amplified during the PCR and whether there has been incorporation of the DIG-labelledd-UTP, run 5 μl of the labelling reactions next to non-labelled PCR products on a 2% agarose gel at 10 V overnight to achieve clear distinction between the bands (Fig. 2).

Following gel electrophoresis, under exposure to UV light the labelled products (lanes 1 and 3, Fig. 2) should appear heavier than the control unlabelled product (lanes 2 and 4, Fig. 2), as a result of high-density labelling with DIG. Purify the labelled products using the Qiagen gel purification kit. Use a total of eight columns for the eight PCR reactions. Elute each column with 30 μl of elution buffer for the neo probe. For the *lacZ* probe, pool four columns at a time in 30 μl of elution buffer, using the eluate of one column as the elution solution for the next. Run an aliquot of purified products again on a 2% agarose gel. Purified probes are quantified using Nanodrop or other spectrophotometry-based method, aliquoted and stored at −20 °C until required.

### 2.4. Digestion of gDNA

Reagents:

Restriction endonucleases and corresponding buffers (FastDigest enzymes from Thermo Fisher Scientific or standard restriction endonucleases from New England Biolabs)

1.0 M Spermidine (omit for FastDigest enzymes)

100x BSA (New England Biolabs)

For each reaction, digest 20–25 μg DNA in a final volume of 85 μl. Set up reactions as follows: 8.5 μl 10x Buffer, 1 μl 0.1 M Spermidine, 1 μl 100x BSA if applicable and 5 μl restriction enzyme. Incubate for 5 h to overnight, according to manufacturer’s instructions. For standard restriction enzymes, spike digests with 1.5 μl restriction enzyme diluted in 13.5 μl 1x Buffer in the morning and digest for a minimum of 3 to 4 additional hours. Freeze down digests until ready to run the Southern blotting process.

### 2.5. Electrophoresis of DNA digest

Reagents:

6x loading dye: 10 mM Tris-HCl (pH 7.6) 0.03% bromophenol blue, 0.03% xylene cyanol FF, 60% glycerol, 60 mM EDTA

10 × TAE: 400 mM Tris, 200 mM acetic acid, 10 mM EDTA

1% ethidium bromide

GeneRuler DNA Ladder (Fermentas, SM0331) known as small ladder, stock adjusted to 15 ng/μl

GeneRuler High Range DNA Ladder (Fermentas, SM1351) known as large ladder, stock adjusted to 15 ng/μl

Seakem® LE agarose (Lonza, 50005)

Day 1: Prepare ladder samples according to the probes to be used. Make enough for 3 wells of small ladder (0.1–24 kb) and 1 well of large ladder (10–48 kb) and store at 4 °C until ready to load:

For *lacZ* gel: small ladder (20 μl small ladder stock, 57 μl 6 × loading dye, 263 μl H_2_O), large ladder (1.5 μl small ladder stock, 28 μl 6 × loading dye, 135 μl H_2_O)

For neo gel: small ladder (72 μl small ladder stock, 57 μl 6 × loading dye, 211 μl H_2_O), large ladder (30 μl small ladder stock, 28 μl 6 × loading dye, 111 μl H_2_O)

The volumes of small and large ladders can be adjusted accordingly depending on the strength of the ladder bands once the Southern has been developed. For example, if the ladder is too weak, increase amount of ladder for the next Southern. If the ladder is too strong i.e. *lacZ* probe has a very strong affinity for the large ladder, use less material.

For a 20 cm × 22.5 cm electrophoresis gel prepare 1 L of 0.45% Seakem® agarose in boiling 1x TAE and cool the solution. Add 40 μl ethidium bromide prior to pouring when solution is warm and leave gel to set in a fume hood.

Defrost restriction digests if required and add a 6th volume (14 μl or 17 μl for spiked digests) of 6x loading dye to each. Once the gel is placed in the tank and is covered by 1x TAE, place the gel tank on magnetic stirrer(s) and place magnetic flea(s) within the tank (do not turn on yet). Remove the comb and load samples. Fill any empty wells with 1x loading dye. Run overnight at 75 V (at 4 °C) or 40 V (at room temperature). Turn on spinners approximately 4 h after loading the gel to circulate the TAE buffer overnight.

### 2.6. DNA transfer to membrane and immobilization

Reagents:

Milli Q water

HCl Hydrolysis solution (2 L per gel): 1978 ml H_2_O, 22 ml fuming HCl.

NaOH pellets diluted to 0.4 M solution in H_2_O (2 L per gel).

Whatman 3 mm chromatography paper (Scientific Laboratory Supplies)

GE Healthcare Amersham Hybond N+ membrane

Day 2: Prepare material for the semi-dry transfer: cut one piece of Hybond N+ membrane to the size of the gel, cutting off the left-hand top corner as the membrane lies curling onto the table. Also cut three pieces of Whatman filter paper 1 to 2 cm larger than the membrane in both dimensions. NOTE: Ensure solutions are prepared prior to visualizing the gel, as dissolving the NaOH pellets can be time-consuming. When the dye front has run at least ¾ of the length of the gel, remove the gel and image on a UV *trans*-illuminator. Digests should appear as smears down the lanes if the DNA has been digested well. Place the gel (still in the gel tray) in a clean rocker tray and wash on the rocker for 10 min with 2 L of HCl hydrolysis solution. Transfer the gel to a clean rocker tray and wash on the rocker for 20 min with 2 L 0.4 M NaOH. Transfer the gel to a clean tray and repeat a second 20 min wash with fresh 2 L 0.4 M NaOH. Take the gel out of the rocker tray and slide off the gel tray onto a clean flat surface (for example another gel tray). Wet the Hybond N+ membrane in MilliQ water and then in the 0.4 M NaOH solution that was used to wash the gel. Place the membrane over the gel with the cut corner at the top right and smooth out any air bubbles between the membrane and the gel. This must be done very gently to avoid damage to the membrane. Wet each piece of filter paper in the 0.4 M NaOH wash and place each on top of the membrane. Stack a layer of ~ 50 folded paper towels packed tightly on top of this. Place a cleaned gel tray and a ~ 1 kg weight on top to assist the transfer and leave 4 h to overnight, as convenient. Ensure that the tray with the weight on top sits evenly on the towels to prevent uneven blotting.

Wash hybridization tubes in MilliQ water and leave to dry overnight.

### 2.7. Hybridization and washes

Reagents:

DIG Easy Hyb buffer (Roche)

20× Saline Sodium Citrate (SSC): 3.0 M NaCl, 0.3 M sodium citrate, pH 7.0

DIG-labelled probe, stored at −20 °C (see section 4.1)

20% Sodium dodecyl sulfate (SDS)

Day 3: Ensure that the waterbath and the two hybridization ovens are at the appropriate temperatures (42 °C for the water bath; 42 °C for the neo probe oven and 40 °C for the *lacZ* probe oven, recorded on a thermometer in a hybridization tube). NOTE: for all wash steps in the rocker trays, add the solution to the tray prior to the membrane to prevent any drying of the membranes during transfer from one wash to another.

Distribute in Falcon tubes 3 × 25 ml DIG Easy Hyb Buffer for each gel (two pre-hybridization washes and one hybridization wash), and place tubes in the waterbath to preheat for at least 20 min before starting the next stage.

Remove the weight, towels and filter paper from the gel and discard. Flip over the gel (which will have lost moisture and shrunk to a thin film) and membrane together and label the position of the gel wells on the membrane using a pencil. Once the wells are marked on the membrane, the gel can be disposed of appropriately. Crosslink the DNA to the membrane by exposing both sides of the membrane to 1200 μJ UV light using a Stratalinker. Place membranes in a clean tray containing 2 L of 2× SSC and wash on a rocker for 5 min. While the membrane is in 2× SSC, place the tray on a slight angle to create a deeper solution at one end of the tray. Fill a clean beaker with the SSC and then gently roll the membrane from bottom to top with the DNA facing inwards (pencil side up) keeping the membrane submerged at all times, and once rolled place in hybridization tube (hold tube with left hand and place membrane in using right hand, so the membrane is not rolled into a tight roll by the hybridization oven motion). Add 2× SSC from the beaker and cover the opened end with a hand or close the tube with the cap before rolling it to unwind the membrane, pressing it to the inside of the hybridization tube.

Fill the tube to the top with 2× SSC and hold the tube upright. This will cause air bubbles between the membrane and the inside of the hybridization tube to migrate upwards and out of the solution. Empty the tube and place the preheated 25 ml DIG Easy Hyb buffer inside. Place the hybridization tube in a 42 °C hybridization oven (for neo probe) or an oven set at 40 °C (for *lacZ* probe), making sure the lid of the tube is on tight and the rotor is balanced. Incubate for approximately 1 h to pre-hybridize.

Repeat the pre-hybridization with fresh, preheated DIG Easy Hyb buffer for an additional 2 h to ensure that all remaining SSC is removed. NOTE: Sealed bags can be used as an alternative to hybridization tubes with smaller volumes of solution but they require careful handling and movement to ensure probe distribution and efficient washes.

Meanwhile, preheat a further 25 ml DIG Easy Hyb buffer in a Falcon tube to 42 °C. Calculate how much DIG-labelled probe is required for 55 ng/ml *lacZ* and 40 ng/ml for neo, in 25 ml of DIG Easy Hyb Buffer. Denature the probe by placing it in a tightly closed tube in boiling water for 5 min, then place tubes straight onto ice for 3 min. Add the required amount of denatured probe to preheated DIG Easy Hyb buffer and invert to mix. Replace the pre-hybridization solution in the hybridization tube with 25 ml of hybridization-probe solution. Hybridize at 42 °C (for neo probe) and 40 °C (for *lacZ* probe) for at least 14 h overnight.

NOTE: Many factors influence the optimal hybridization temperature for each combination of probe and hybridization buffer, including buffer recipe and the GC content and length of probes. The optimal temperatures were determined empirically and also take into account the potential variations in the calibration and performance of hybridization ovens.

Prepare wash solutions ready for the next day according to the probes to be used, taking care to avoid any precipitation of chemicals in solutions.

For neo probe:

2× 2 L 2× SSC, 0.1% SDS: leave SSC at room temperature overnight and add 10 ml of SDS (incubated separately at 37 °C overnight) the following morning.

2× 2 L 0.2× SSC, 0.1% SDS: incubate at 65 °C overnight.

For *lacZ* probe:

2× 2 L 2× SSC, 0.1% SDS: incubate each component separately at 37 °C overnight and mix together the following morning.

2× 2 L 0.1× SSC, 0.1% SDS: incubate at 65 °C overnight.

Day 4

Reagents:

5× maleic acid: dissolve powder in warm water, adjust pH to 7.5 using 5 M NaOH, make up volume to 2 L and filter if necessary.

1 L 5 M NaCl

10× BBR Block – 1 g BBR Blocking Reagent (Sigma-Aldrich) and 10 ml 1× maleic acid (diluted 1:5 with H_2_O). If making aliquots, add 25 g of BBR to 50 ml of maleic acid and 200 ml H_2_O. Make 10 ml aliquots and store in freezer. Aliquots can be placed in 42 °C water bath to speed up defrosting.

Buffer I (4 × 2 L) 1800 ml H_2_O, 200 ml 10x TBS, 6 ml Tween 50/50 (Tween 20 1:2 in H_2_0)

Buffer III (2 L) 1860 ml H_2_O, 100 ml 1 M Tris HCl pH 9.5, 40 ml 5 M NaCl

Block (20 ml per membrane) – 18 ml Buffer I, 2 ml 10x BBR

Antibody (20 ml per membrane; store at 4 °C until needed, prepare shortly before use) 18 ml Buffer I, 2 ml 10× BBR, 2 μl Anti-Digoxigenin-AP Fab Fragments (used at 1:10,000)

CDP-Star (15 ml per membrane) 15 ml Buffer III, 50 μl CDP-Star (i.e. used at 1:300)

Put the appropriate wash solution for the probe used into a clean tray on the rockers. Take the hybridization tubes from oven, remove the membranes and put into wash trays in accordance with the probe used, ensuring that the membrane is unrolled once in the solution. Multiple membranes for analysis with the same probe can be washed together. Wash the membrane(s) for 10 min at room temperature in 2 L 2× SSC, 0.1% SDS, stored at room temperature (for neo) or stored at 37 °C (for *lacZ*). Repeat this wash step in a clean tray. Ensure that the membranes are covered well by the solution and do not fold up as they rock. Transfer the membranes to clean trays of solutions and wash on rockers for 30 min in 2 L 0.2× SSC, 0.1% SDS stored at room temperature (for neo) and 0.1× SSC, 0.1% SDS incubated at 65 °C (for *lacZ*). Repeat this wash step in a clean tray.

Meanwhile, make up 8L of Buffer I for one to four membranes and leave covered in a cylinder on the bench. From this point, membranes can be washed together in the same tray irrespective of the probe used. Add 2 L Buffer I to a clean tray, submerge the membrane(s) and wash on the rocker for 5 min to equilibrate. Roll the membrane up as before and place individually into a clean hybridization tube. Add 20 ml of block to the tube and incubate in a hybridization oven at room temperature (minimum 20 °C) for 30 min. After blocking, remove the hybridization tube from the oven, pour out the block and add 20 ml of antibody solution to the tube. Incubate in a hybridization oven at room temperature (minimum 20 °C) for 30 min. Following antibody incubation, remove the membrane and place in a clean tray. Wash on a rocker for 10 min in 2 L of Buffer I. Transfer the membrane to a clean tray and wash on a rocker for 20 min in 2 L of Buffer I. Transfer the membrane to a clean tray and wash for ~ 1.5 h in 2 L of Buffer I. The tray can be left flat on the bench for this long wash step, making sure the membranes remain fully submerged at all times. Prepare buffer III solution as the 1.5 h incubation step comes to an end. Transfer membrane to a clean tray and wash on a rocker in Buffer III for 5 min to equilibrate.

### 2.8. Antibody staining and signal detection

Cut a sheet of plastic film so that it is slightly larger than the membrane. Open the plastic sheet, place the membrane into the crease and fold the plastic back over. Push out any air bubbles very gently with folded soft paper towels or tissues, as too much pressure can damage the membrane. Seal the plastic twice around each of the three open edges using a heat sealer, keeping the plastic as flat as possible, so that the membrane is completely sealed inside the plastic. Cut open a corner of the plastic bag and add approximately 13 ml of the CDP-Star chemiluminescent substrate solution. Gently ensure that air bubbles are pushed out before re-sealing the plastic and placing on a rocker for 5 min. Once incubated, cut another corner of the plastic bag and discard the majority of the CDP-Star from the bag. Then, remove as much of the CDP-Star solution as possible using a roller and/or tissue and re-seal the bag carefully around the membrane. For chemiluminescent detection of the signal, use a BioRad ChemiDoc™ Touch Imaging System, according to the manufacturer’s instructions or autoradiography. NOTE: When using a lower amount of gDNA per lane, the signal intensity can be improved by pre-incubating the CDP-Star-exposed membrane for 1 h at 37 °C before starting signal acquisition.

When using a ChemiDoc™ system for visualisation, place the membrane with the DNA facing up on the tray. Adjust the position of the membrane to ensure full exposure. Membranes can be secured with tape. Select ‘Live View’ and adjust the image size to large. Under ‘Application’ select ‘Blots’ and ‘Chemiluminescence’. Under ‘Exposure’ select ‘Manual’ and ‘Configure Signal Accumulation Mode’. Set the first image to 600, the last image to 3600 and the number of images to 6. This will take one image every 10 min for 1 h. Press the exposure link. After the images are taken, they will appear in the gallery and must then be saved. Select the images (usually the 30 min exposure) and adjust the brightness/contrast. Export the images as a .jpg file. The image can then be printed to measure band sizes.

NOTE: To strip membranes that were hybridized with alkali-labile probes it is possible to wash them with an alkaline buffer (0.2 M NaOH/0.1%SDS) twice for 15 min at 37 °C and repeat the process starting at pre-hybridization.

### 2.9. Differences with a classical protocol using radioactive probes

Radioactively-labelled probes were used prior to the development of non-radioactive probes, with the differences to the overall protocol presented in the following stages: probe preparation, choice of DNA molecular weight standard, hybridization and washes, and signal detection. Appropriate use of personal protective equipment, shielding and chemical disposal management must be adopted when using radioactive chemistry.

#### 2.9.1. Radioactive probe synthesis and purification

Reagents:

Megaprime DNA labelling System, dCTP (Amersham)

α−^32^P dCTP (37 MBq (ImCi; PerkinElmer). Note that this is a hazardous chemical. Appropriate precautions (as per applicable regulations) must be taken at all steps involving this radioactive compound.

Nick-columns 50ST (GE Healthcare)

TE: Tris-HCl 10 mM (pH7.5–8.0), disodium EDTA 1 mM

Monocut lambda DNA molecular weight 0.5 μg/μl (New England Biolabs)

The DNA fragment to be labelled is obtained by PCR with standard primers. The PCR product is purified and quantified using Nanodrop or another spectrophotometry-based method. Preheat a water bath at 100 °C. Prepare the following Eppendorf tubes: for each probe, include 5 μl of random primers, 27 μl H_2_O and 1 μl probe at 25 ng/μl. Place the reactions at 100 °C for 5 min, then place on ice for 5 min, centrifuge rapidly, then keep on ice. On ice, mix together 10 μl labelling buffer and 2 μl Klenow per reaction and distribute 12 μl mix to each reaction. In a radioactive area, add 5 μl α−^32^P dCTP (total volume: 50 μl) and incubate for 30–45 min at 37 °C. The probe can be immediately purified or stored at −20 °C until purification.

For probe purification, equilibrate a Nick column with 3 ml of TE. Place the column on an Eppendorf Safe-Lock 2 ml tube, and load 50 μl of labelled probe. Add 400 μl of TE and let flow through. Place the column on a new 2 ml Safe-Lock tube and add 400 μl TE. Collect the purified probe (check labelling with counter or count 1 μl). Check incorporation of radioactive nucleotides by waving the tube next to a Geiger counter. Probes can be stored at −20 °C or at 4 °C if it is to be used on the same day.

#### 2.9.2. Choice of DNA molecular weight standard

When using radioactive probes, for neo gels include three wells containing 3 μl GeneRuler DNA ladder, 1 μl monocut lambda 0.5 μg/μl (NEB) and 26 μl Blue 1× and one well with 10 μl GeneRuler High Range DNA and 20 μl 1× loading dye.

#### 2.9.3. Pre-hybridization, hybridization and washes

Reagents:

Rapid Hyb Buffer (GE Healthcare)

Membranes may be dried and stored between filter paper at 4 °C for several days prior to the hybridization step. Preheat to 65 °C the Rapid Hyb Buffer (10 ml per probe). Prepare the membranes after transfer: label the ladder bands and wells with a pencil; cut with a scalpel and immerse in distilled water. Transfer the membranes (with wells at the bottom of the tube and DNA side facing inwards; the addition of an additional DNA-free membrane facing the DNA side drastically decreases background noise) to a roller containing hybridization buffer preheated to 65 °C. Incubate for 20 min to 1 h at 65 °C in the hybridization oven. Thaw the radioactive probe if previously frozen. Denature the probe for 5 min at 95 °C in a heating block and transfer the denaturated probe in a hybridization tube, taking care not to touch the membrane. Hybridize for 3 h or more (or overnight if more convenient) in a hybridization oven.

The following washes are performed with 50 ml of each solution in hybridization tubes at 65 °C: two with 2× SSC; 0.1% SDS, each for 15 min; two with 0.1× SSC, 0.1% SDS each for 15 min. The last two washes can be performed in a tray under agitation. Scan the membranes with a radioactive counter (Canberra MC21 type); the signal must be slightly audible. If the signal is too strong, perform an additional wash.

#### 2.10.4. Signal detection

Reagents:

Kodak BioMax MR 30 × 40 cm or 24 × 30 cm autoradiography films

Blot the membranes on paper towels and place in a plastic bag cut to the dimensions of an autoradiographic film; place in an autoradiography cassette with two screens. Place an autoradiography film shiny face upwards in the dark room; mark the film orientation at a corner. Store the cassette at −80 °C for two days (adapt the time and temperature to the radioactive signal intensity after washes).

Probes can be washed off prior to hybridization with other probes if the membranes have not been dried off.

## 3. Results analysis and interpretation

**Fig. 3.**
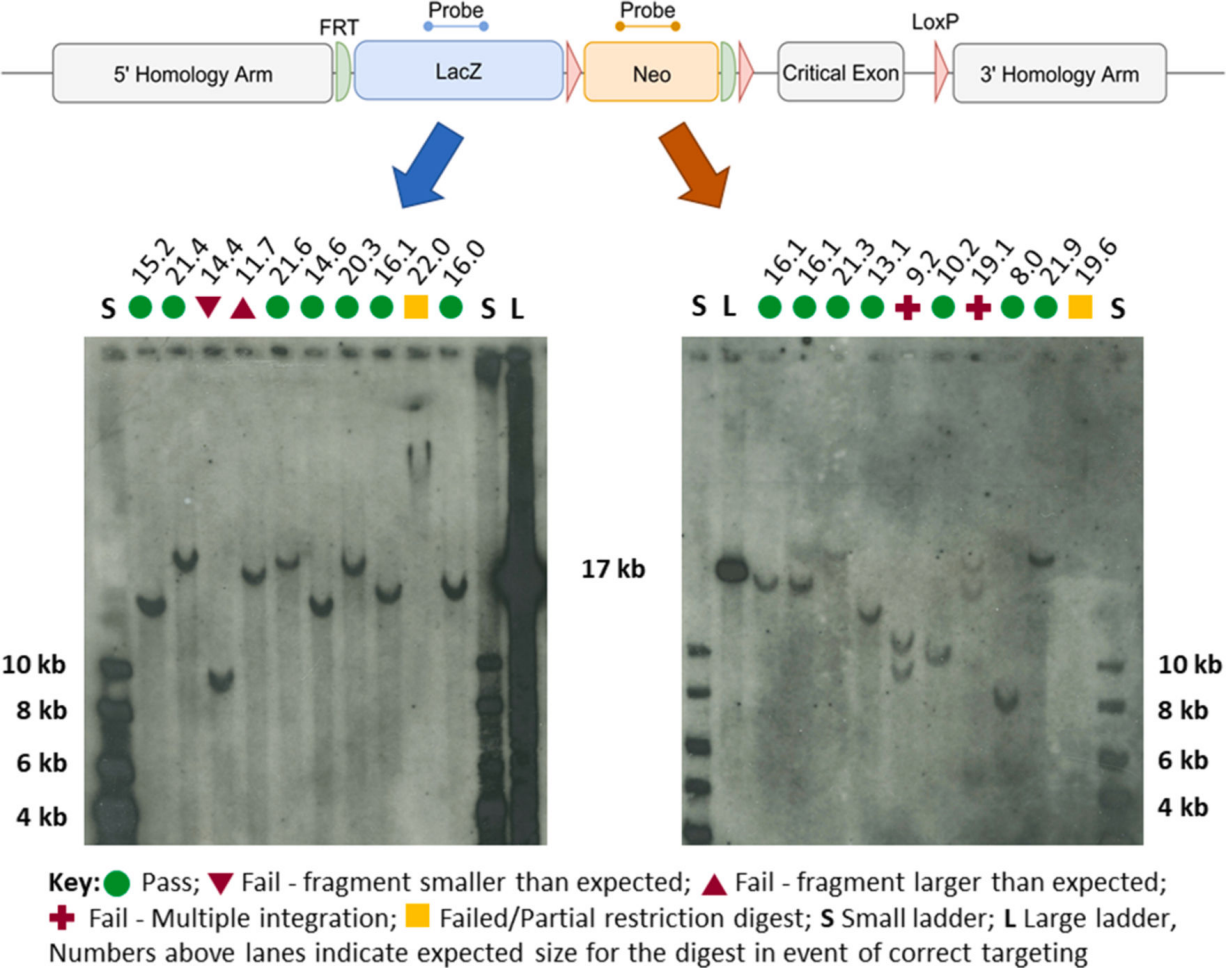
Example of Southern blot results obtained with DIG-labelled probes. A, Map of a typical tm1a targeted allele [1] with position of *lacZ* (blue) and neo (red) probes. B, Examples of Southern blot autoradiographs obtained using either the *lacZ* probe (left hand side) or neo probe (right hand side) of tm1a ES cell clones. Each blot has two size standards; a small ladder (S) and a large ladder (L), with their sizes indicated in bold. The expected size (kb) of each of the visualised fragments is displayed above each of the lanes. The interpretation of each line is indicated by symbols as per the key.

Typically, two or three restriction enzymes (see design in Fig. 1), each designed to generate a DNA fragment that encompasses the probe segment and an entire homology arm, are chosen for each side of the targeting event. Fig. 3 shows typical examples of Southern blot results obtained with DIG-labelled probes and gDNA extracted from EUCOMM/KOMP clones bearing tm1a alleles [1]. According to the enzymes applied, each set of restriction enzymes has an expected pattern that is specific to a correctly targeted locus. The size standards 17 kb, 10 kb and 8 kb bands are recognised by the probes. Comparison to migration patterns of the molecular weight ladders allows for an estimation of the size of each band.

The profiles of band sizes that are obtained with the different restriction enzymes are compared to the pattern expected (“Expected sizes” shown above membrane pictures in Fig. 3). The actual size of each positive band is compared to its expected value. Green circles mark lanes with bands of expected sizes in Fig. 3 and correspond to a “Pass” data point. Red triangles pointing down or pointing up mark positive bands of smaller or larger than the expected size, respectively, and correspond to a “Fail” data point. Note that in most cases only one band was obtained per lane with minimal background noise (see whole membrane lengths in Supplemental Figs. S1 and S2). A clone is validated if at least two digests generating a band that encompasses each homology arm of the targeting event shows the correct pattern (“Pass”); that is, two data points per homology arm, totalling four. If two separate restriction digestions yield an unpredicted pattern, the clone is not brought forward for chimera generation. Multiple bands indicate that the targeting construct has inserted several times into the genome. However, they may also be due to partial gDNA digestion or enzyme star activity. Multiple insertions are therefore conclusively ascertained if shown by more than one enzyme or enzyme combination. A band of a very large size (> 20 kb; for example, in lane marked by yellow square on left-hand membrane in Fig. 3) may be down to partial digestion or an aberrant recombination event. The absence of a band (for example, in lane marked by yellow square on right-hand membrane in Fig. 3) is either due to insufficient gDNA loaded in a well, severe allele rearrangement or failure to digest. Very large bands and absence of a band are therefore inconclusive data points. Clones presenting three “Pass” and one “Fail” data points are tested with additional restriction enzymes, if no other clone with an entirely correct pattern corresponding to the same project is available. Particular attention is given to the possibility of inhibition of digestion by gDNA methylation when partially failed profiles are interpreted. In such cases, additional restriction digests with other enzymes are performed.

**Fig. 4.**
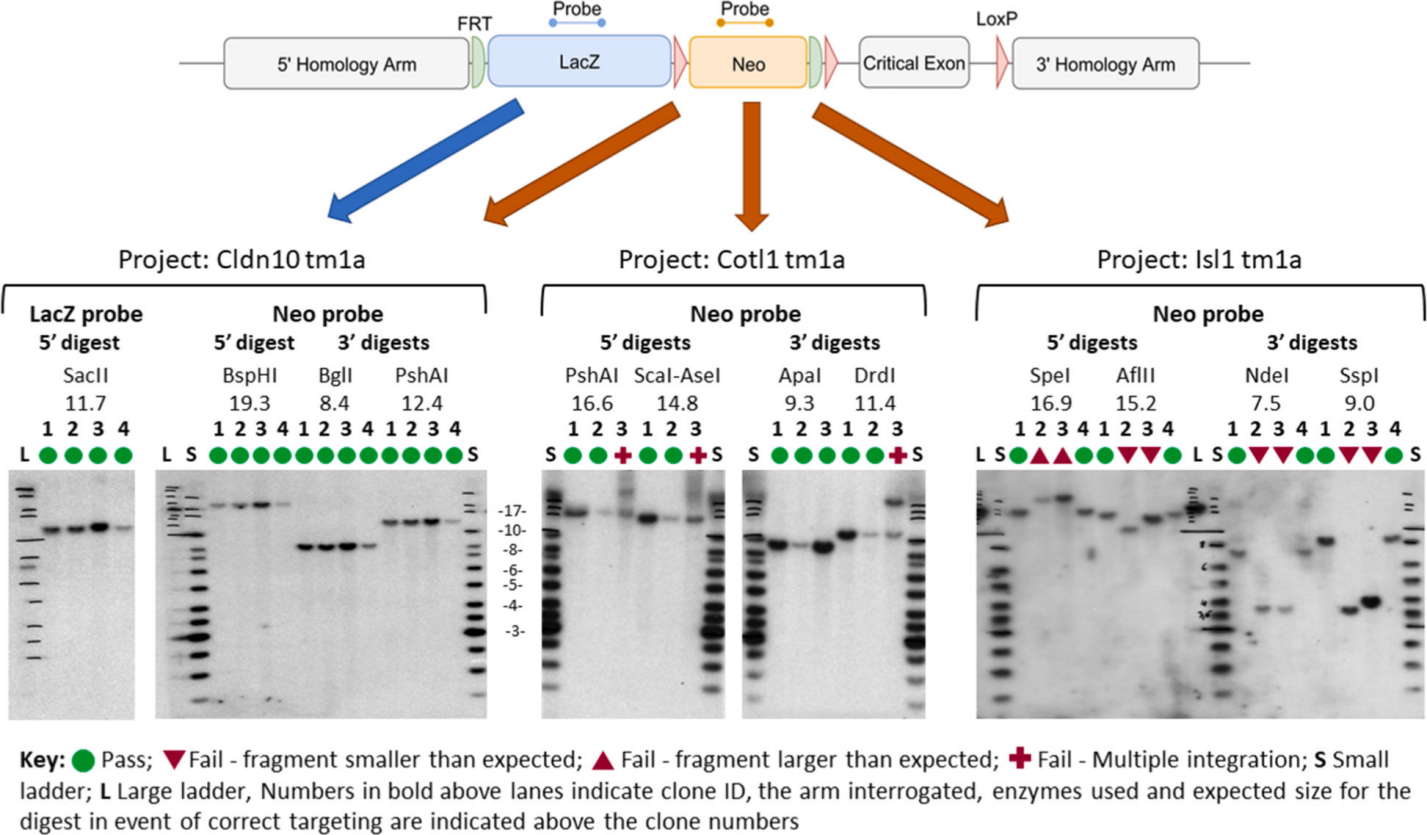
Example of three projects (Cldn10, Cotl1 and Isl1) analysed by Southern blot using radioactive probes. For each project, three or four clones have been processed. A *lacZ* probe was used only when no acceptable 5′ restriction size (> 20 kb) was possible (Cldn10 project). For the Cotl1 project, the 3′ ApaI digest for clone 3 does not show a double band, whilst other digests do. This suggests that two bands of the same size are generated by this enzyme.

Fig. 4 shows an example of Southern blot results obtained with radioactive probes and gDNA extracted from EUCOMM/KOMP clones. *LacZ* and/or neo probes are employed depending on allele restriction maps. Clones showing the expected band pattern (that is, clones 1–4 for project Cldn10, clones 1 and 2 for project Cotl1, and clones 1 and 4 for project Isl1 Fig. 4) are taken forward to chimera generation. Clones displaying aberrant patterns (that is, clone 3 for project Cotl1, and clones 2 and 3 for project Isl1, Fig. 4) are discarded. A similar overall picture for the validation of clones for the Nras project obtained with DIG-labelled probes is shown in Supplemental Fig. S3.

## 4. Discussion

The protocol represents a lengthy process that requires careful execution of multiple steps but which generates, when at least two different digests for each homology arm are used, a survey of the integrity of the structure of an allele modified by homologous recombination (Fig. 1). One can observe different and non-overlapping band patterns when the same restriction enzyme and probe combination is applied to different clones, showing that no recurrent (non-specific) signal occurs (Supplemental Fig. S3).

The switch from radiolabelled to digoxygenin-labelled probes represents an important gain in terms of safety for the personnel involved in the execution of the protocol. The method presented retains sufficient sensitivity to obtain a signal detectable from a single copy integration, even if the signal to noise ratio may be smaller than with the radioactive equivalent (See Supplemental Fig. S1 for the whole length of the membranes). However, the method requires specific adjustments from a classical radioactivity-based protocol (2.9) and its implementation requires more laboratory work at the stage of signal detection. It is also essential that special care is taken to ensure that membranes and trays are not handled with bare hands or powdered gloves and that all instruments are cleaned and abundantly rinsed with water to prevent accumulation of background noise.

Working with universal internal probes bypasses the need for a time-consuming and labour-intensive optimisation of locus-specific external probes. Instead, the combination of restriction enzyme digests (two for each homology arm) defines a profile that documents the integrity of a targeted event (Figs. 1 and 4 and Supplemental Fig. S3). Finding enzymes that generate acceptable DNA fragment sizes (< 20–25 kb) may be difficult for some alleles. For these cases, a double digest with two restrictions enzymes can be employed in combination with internal probes. However, external probes also serve to ascertain the map of the endogenous locus and comparison between the intensities of the WT and targeted bands informs on the possibility of mixed clones containing both WT and targeted ES cells. Alternatively, this parameter can be monitored by loss-of-allele qPCR assays [9].

This Southern blot protocol can be utilised for material validation after production or importation from a repository of targeted ES cell clones and prior to chimera generation. This is a generic protocol applicable to all alleles targeted in ES cells that contain standard transgenic cassettes, including all knockout-first alleles [1]. Only the specific restriction enzymes to be applied need to be modified for each specific allele to be tested in accordance with the allele sequence, without the requirement of time-consuming optimization of experimental conditions for project-specific sets of probes.

We have presented a protocol based on the digestion of a generous amount of gDNA (20–25 μg) for stability of the process. This accommodates some variability in the quality of the gDNA, which is particularly useful in large-scale experiments and provides a sensitive assay to detect mixed ES clones that can arise in large-scale production of targeted libraries. It is possible to work with a smaller amount (5 μg) and downsize all surface area and volumes accordingly for a more economical protocol or when working with limited sources of tissues. In this case, exposure of membrane should be lengthened accordingly.

However, it is beneficial to pre-validate clones with other assays that frequently eliminate clones from the pipeline and are less time-consuming and expensive to implement. Moreover, Southern blot assays are not sufficient to entirely validate ES cells prior to injection; the presence of a distal loxP site must be ascertained by PCR [2] and euploidy of the clone should be verified [10]. The order and extent of validation tests to run prior to Southern blotting will depend on the processes available in the laboratory: for example, if karyotypic assessments are executed by ddPCR chromosome counting, the test can be implemented at an early stage of the process (and with less ES cell material).

However, if the test is implemented with chromosome spreads, this assay is more practically performed as a final validation step of the colonies prior to chimera generation, due to its expense and requirement for a highly-trained operator [10].

Validation of ES cells that includes Southern blot assays of the targeted allele with internal probes, and where four enzymes are found to yield correct patterns, prevents the generation of animals with improper targeting that will need to be culled. Such an approach supports the 3Rs requirement of minimizing the number of animals used in research. However, ES cell colonies may not be homogeneous, and a clonal event can occur at the point of fertilization of eggs by a single sperm for germline transmission. Therefore, the process does not provide a definitive assurance that the allele transmitted to the offspring of the chimera is correct.

Importantly, a validation process based upon Southern assays is a better predictor of transmission of a validated allele than those strategies based entirely on PCR and qPCR [2]. In the case of knockout-first alleles, the identity of the modified locus and the presence of loxP sites distal to the targeted locus should be ascertained by the amplification and verification by sequencing of a PCR product containing both loxP sites, prior to Southern blotting, as these are cheaper and less time-consuming assays that will eliminate a significant number of ES cell clones. For comprehensive validation of mouse stock, the presence of the loxP sequence distal to the selection cassette should be ascertained in F1 mice by PCR amplification and sequencing. In addition, genotyping of mice performed by cassette copy counting [11] and loss-of-allele assay [9] provides greater assurance of the accuracy of the genetic characterisation of the animals.

## 5. Conclusion

We present a Southern blot protocol that relies on universal probes to avoid the requirement of project-specific probes and employs non-radioactive labelling for enhanced safety for the operator. Validation of the overall structure of homologous recombination events is essential to ensure the quality of newly generated or imported targeted mouse lines.

## Supplementary Material

1

## Footnotes

1 ES: Embryonic Stem; HA: homology arm; IMPC: International Mouse Phenotyping Consortium; IAA: Isoamyl alcohol.
