# Supplementary material for: Universal Southern blot protocol with cold or radioactive probes for the validation of alleles obtained by homologous recombination
Source: Methods. Author manuscript; Available in PMC 2024 Jan 16. (PMC10790599; doi:10.1016/j.ymeth.2020.06.011)
Supplement: 1 [file NIHMS1620012-supplement-1.pptx]

## Slide 1
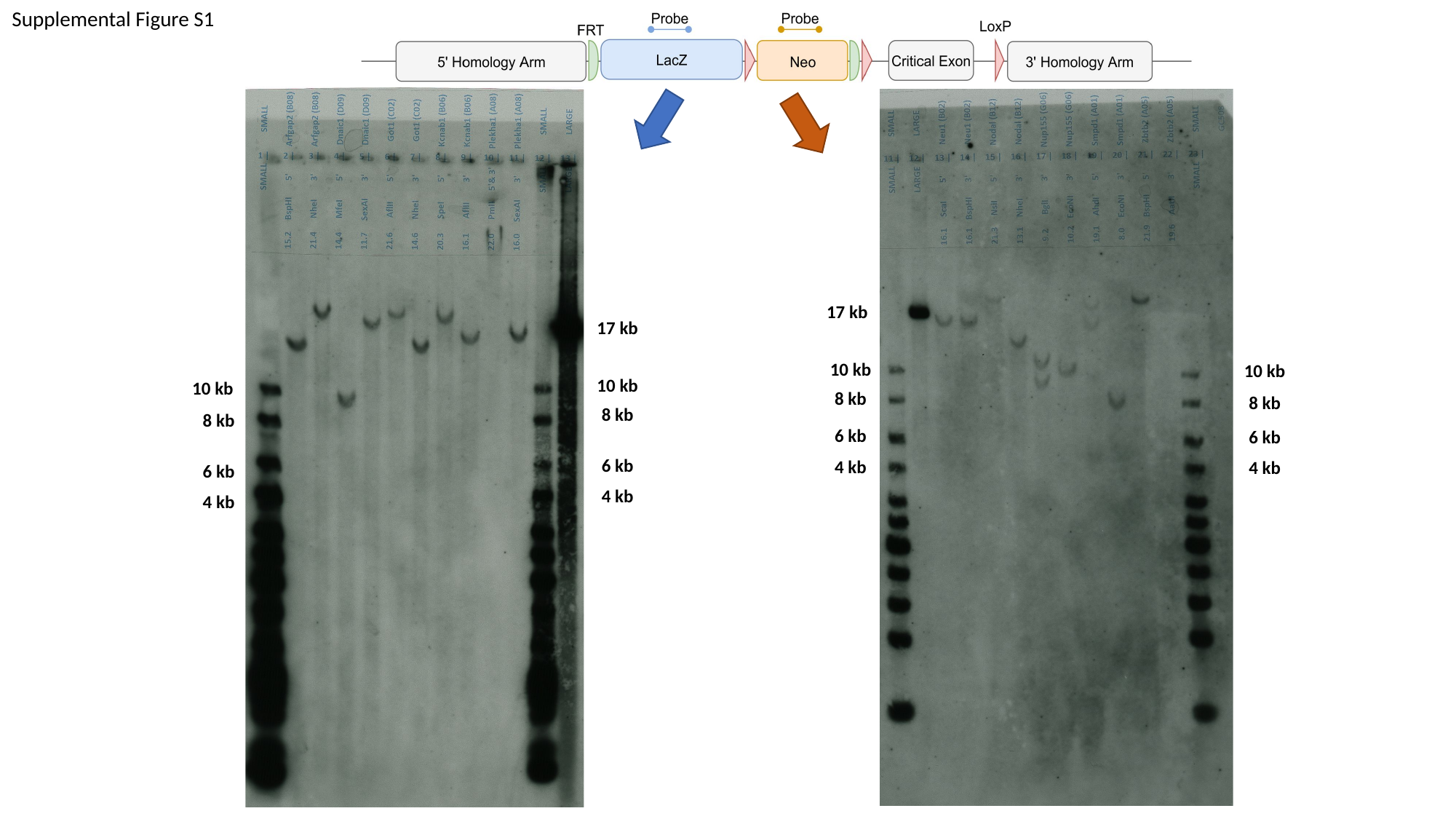

Supplemental Figure S1
17 kb
10 kb
10 kb
8 kb
8 kb
6 kb
6 kb
4 kb
4 kb
17 kb
10 kb
10 kb
8 kb
8 kb
6 kb
6 kb
4 kb
4 kb

## Slide 2
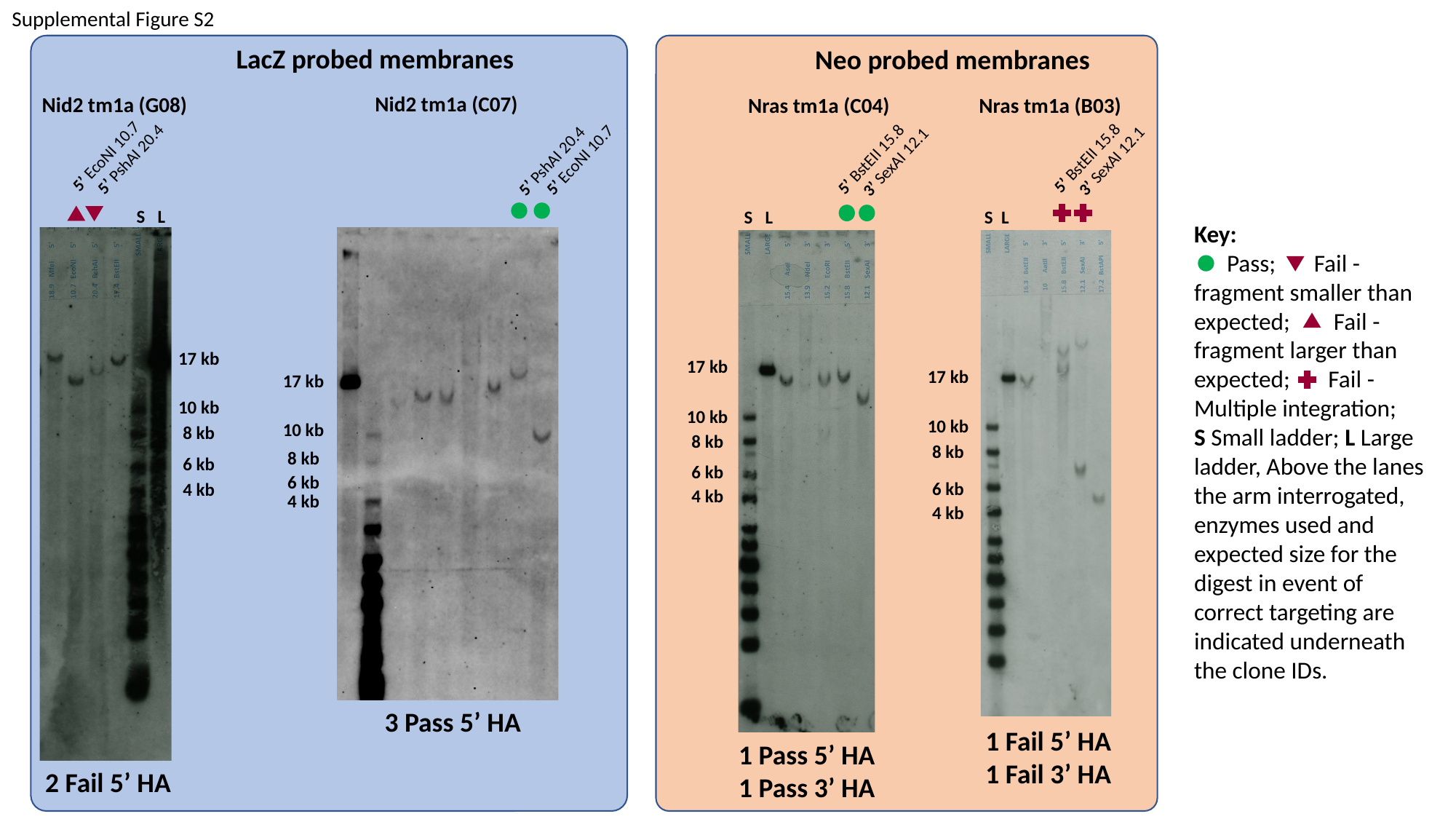

Supplemental Figure S2
LacZ probed membranes
Nid2 tm1a (C07)
 5’ PshAI 20.4
 5’ EcoNI 10.7
17 kb
10 kb
8 kb
6 kb
4 kb
5’ EcoNI 10.7
 5’ PshAI 20.4
S L
17 kb
10 kb
8 kb
6 kb
4 kb
Nid2 tm1a (G08)
Neo probed membranes
Nras tm1a (C04)
5’ BstEII 15.8
 3’ SexAI 12.1
S L
17 kb
10 kb
8 kb
6 kb
4 kb
Nras tm1a (B03)
S L
17 kb
10 kb
8 kb
6 kb
4 kb
5’ BstEII 15.8
 3’ SexAI 12.1
1 Fail 5’ HA
1 Fail 3’ HA
1 Pass 5’ HA
1 Pass 3’ HA
Key:
 Pass; Fail - fragment smaller than expected; Fail - fragment larger than expected; Fail - Multiple integration;
S Small ladder; L Large ladder, Above the lanes the arm interrogated, enzymes used and expected size for the digest in event of correct targeting are indicated underneath the clone IDs.
3 Pass 5’ HA
2 Fail 5’ HA

## Slide 3
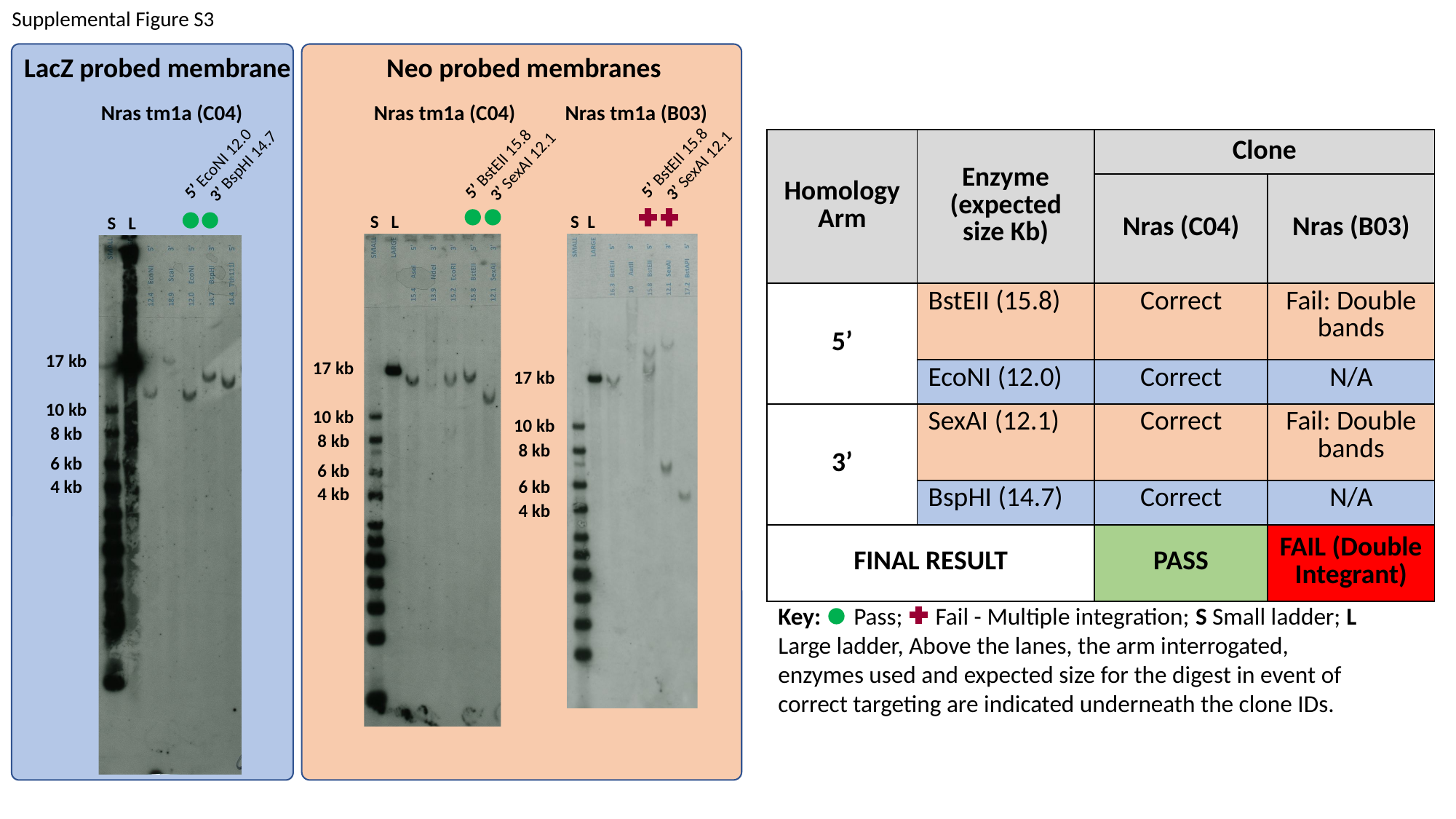

Supplemental Figure S3
LacZ probed membrane
Nras tm1a (C04)
5’ EcoNI 12.0
 3’ BspHI 14.7
S L
17 kb
10 kb
8 kb
6 kb
4 kb
Nras tm1a (C04)
5’ BstEII 15.8
 3’ SexAI 12.1
S L
17 kb
10 kb
8 kb
6 kb
4 kb
Nras tm1a (B03)
S L
17 kb
10 kb
8 kb
6 kb
4 kb
5’ BstEII 15.8
 3’ SexAI 12.1
Neo probed membranes
| Homology Arm | Enzyme (expected size Kb) | Clone | |
| --- | --- | --- | --- |
| | | Nras (C04) | Nras (B03) |
| 5’ | BstEII (15.8) | Correct | Fail: Double bands |
| | EcoNI (12.0) | Correct | N/A |
| 3’ | SexAI (12.1) | Correct | Fail: Double bands |
| | BspHI (14.7) | Correct | N/A |
| FINAL RESULT | | PASS | FAIL (Double Integrant) |
Key: Pass; Fail - Multiple integration; S Small ladder; L Large ladder, Above the lanes, the arm interrogated, enzymes used and expected size for the digest in event of correct targeting are indicated underneath the clone IDs.
